# Postoperative Long-Term Independence Among the Elderly With Meningiomas: Function Evolution, Determinant Identification, and Prediction Model Development

**DOI:** 10.3389/fonc.2021.639259

**Published:** 2021-03-05

**Authors:** Haoyi Li, Huawei Huang, Xiaokang Zhang, Yonggang Wang, Xiaohui Ren, Yong Cui, Dali Sui, Song Lin, Zhongli Jiang, Guobin Zhang

**Affiliations:** ^1^ Department of Neurosurgery, Beijing Tiantan Hospital, Capital Medical University, Beijing, China; ^2^ Department of Critical Care Medicine, Beijing Tiantan Hospital, Capital Medical University, Beijing, China

**Keywords:** independence, meningioma, the elderly, functional evolution, prediction model

## Abstract

**Background:**

Maintenance of postoperative long-term independence has value for older adults who undergo surgical procedures. However, independence has barely caught attention for the elderly with meningiomas. Preventing postoperative long-term independence decline in this population necessitates the identification of the factors related to this outcome and minimizing their implications. Therefore, we assessed the independence evolution and identified potential determinants and population.

**Materials and Methods:**

From 2010 to 2016, elderly meningioma patients (≥65 years old) undergoing operation at Beijing Tiantan Hospital were included in our study. The primary outcome was 3-year (*i.e*., long-term) postoperative independence measured by Karnofsky performance scale (KPS) score. We used univariate and multivariate analyses to determine the risk factors for postoperative long-term independence, and nomogram was established.

**Results:**

A total of 470 patients were included eligibly. The distribution in each KPS was significantly different before and 3 years after resection (*P* < 0.001). Especially in patients with preoperative KPS 80 and 70, only 17.5 and 17.3% of the patients kept the same KPS after 3 years, and the remaining patients experienced significant polarization. The most common remaining symptom cluster correlated with postoperative long-term independence included fatigue (R = −0.795), memory impairment (R = −0.512), motor dysfunction (R = −0.636) and communication deficits (R = −0.501). Independent risk factors for postoperative long-term non-independence included: advanced age (70–74 *vs.* 65–69 OR: 2.631; 95% CI: 1.545–4.481 and ≥75 *vs.* 65–69 OR: 3.833; 95% CI: 1.667–8.812), recurrent meningioma (OR: 7.791; 95% CI: 3.202–18.954), location in the skull base (OR: 2.683; 95% CI: 1.383–5.205), tumor maximal diameter >6 cm (OR: 3.089; 95% CI: 1.471–6.488), nerves involved (OR: 3.144; 95% CI: 1.585–6.235), high risk of WHO grade and biological behavior (OR: 2.294; 95% CI: 1.193–4.408), recurrence during follow-up (OR: 10.296; 95% CI: 3.253–32.585), lower preoperative KPS (OR: 0.964; 95% CI: 0.938–0.991) and decreased KPS on discharge (OR: 0.967; 95% CI: 0.951–0.984) (*P* < 0.05). The discrimination and calibration of the nomogram revealed good predictive ability (C-index: 0.810).

**Conclusion:**

Elderly meningioma patients might present significant polarization trend in maintaining long-term independence after surgery. Our findings will be helpful for guiding surgical management for the elderly with meningioma and provide proposals for early functional rehabilitation.

## Introduction

Meningiomas are the most common primary intracranial tumor, with rising incidence in patients aged 65 years and older (1). The majority of meningiomas are benign and are considered surgically cured once tumor resection is complete (2). Therefore, measures of treatment success in such patients have appropriately shifted to more patient-centered metrics (3), including postoperative quality of life (QoL). Up to 35% of meningiomas are biologically aggressive or surgically inaccessible and have significant risk of recurrence, resulting in a second brain injury and a clinical course of repetitive debilitating treatments (4). Hence the postoperative long-term QoL of these patients should be paid more attention. However, the QoL after surgical resection of tumor in meningioma achieved inconsistent results. The majority of meningioma patients were reported to have improved QoL after surgical resection of tumors (5–7). However, the recent largest prospective longitudinal study of long-term Health-Related Quality of Life (HRQoL) outcomes in meningioma patients found that meningioma patients have sustained clinically significant impairments in global HRQoL after tumor resection (3). As we have known, elderly people are vulnerable to poorer outcomes because of a decrease in physiological reserve and in the ability to deal with stressors due to a systemic decline in health (8). Therefore, the risk/benefit ratio for neurosurgical treatment increase with age (9, 10). However, until now, few studies have investigated the QoL after tumor resection in elderly meningioma patients. Fried et al. reported physical deterioration as part of attenuated QoL was listed as one of the four outcomes that older adults considered as unacceptable results after surgical treatment (11). More recently, in a qualitative study published by Lindsey M. and colleagues, the need to measure long-term independence in the elderly after surgery remains vital due to more than 20% rate of functional decline at 30 days (12). Investigators have acknowledged that long-term maintenance of independence has recently been considered to be the preferred outcomes in older surgical patients in clinical trials (13–15), while, in patients with brain tumors, long-term independence is more likely to reflect better postoperative cognition and less neurological deficits (12, 16–18). Nevertheless, postoperative long-term independence in the elderly with meningioma has barely caught attention.

In this retrospective study, we investigated the incidence of postoperative long-term independence, explored the suspected risk factors that may contribute to the risk of loss of independence after tumor resection in elderly meningioma patients, and developed the risk model for predicting the postoperative long-term non-independence.

## Materials and Methods

### Participants

This was a single-center retrospective cohort study that was conducted in the Department of Neurosurgery at Beijing Tiantan Hospital, Capital Medical University, Beijing, China, from January 2010 to December 2016. Ethical approval was obtained from the Ethics Committee of Beijing Tiantan Hospital, and this study was conducted in accordance with the principles of the declaration of Helsinki.

Elderly patients who were aged 65 or older, pathologically diagnosed with meningiomas and underwent surgical resection of tumor from January 2010 to December 2016 were included. The exclusion criteria were as follows: (i) patients with other brain or spine lesions; (ii) patients with neurofibromatosis type 1 or 2; (iii) concurrence with other malignancies or death from other lethal diseases in hospital or discharge; (iv) concurrence with other diseases affecting postoperative long-term functions after discharge; (v) loss of follow-up data. **Supplementary Figure 1** shows the study flowchart.

### Data Collection

Baseline and clinical data were extracted from hospital electronic medical records (details in **Supplementary Table 1**). Radiology features were evaluated from neuroimaging data by two neuroradiologists who had over 10 years of experience. The World Health Organization (WHO) grade and biological behavior of tumors according to 2016 WHO classification of Tumors of the Central Nervous System (4) were determined based on the pathological reports by two experienced neuropathologists (**Supplementary Table 2**). The extent of resection was extracted from surgical reports which were categorized as gross total resection (GTR) or subtotal resection (STR), according to the Simpson grade (Simpson I–III for GTR, Simpson IV–V for STR) and the postoperative neuroimaging evaluation (2). The tumor shape was defined as regular or irregular, *e.g.*, in terms of mushroom-like growth. Tumor recurrence was defined as an appearance of new contrast-enhancing lesions on MRI after surgery. Lastly, Symptom-related function deficits were collected and categorized as none, mild, moderate, and severe status, and the definition and detailed information are described in **Supplementary Table 3**. Moreover, all Karnofsky performance scale (KPS) scores of individuals were measured before surgery, on discharge, and in postoperative long-term periods.

KPS score has become a standard assessment tool of comprehensive function performance widely used by clinicians and researchers in the neuro-oncology field (19, 20), which is defined in terms of the ability to carry out daily activities for patients. The KPS consists of 11 categorical ratings in increments of 10 that range from 0 (dead) to 100 (normal, no complications; no evidence of disease) (20). KPS from 100 to 0 represents the decreased function outcomes in turn. Furthermore, KPS ranging from 100 to 80, 70 to 50 and 40 to 0 represent independent, partial independent, and dependent daily activities, respectively (21). The cutoff value for KPS was set to 70 based on independence and non-independence (*i.e*., dichotomized as >70 and ≤70) (21, 22).

### Follow-Up

As previously reported in published literature, a large proportion of patients showed spontaneously improved function over the first 1 to 2 years after surgical resection of the meningioma (7), which might suggest the possibility that existing function deficits would begin to improve 3 years later remains slim. Therefore, the follow-up endpoint was defined as 3 years after discharge (*i.e.*, long-term independence). To estimate postoperative long-term functional outcomes more accurately, details about the functional outcomes and daily activities including remaining symptoms which refer to European Organization for Research and Treatment of Cancer Quality of Life Questionnaire-Brain Neoplasm 20 (QLQ-BN20) (23, 24), objective KPS and comprehensive satisfaction for the patients’ life condition using modified Common Toxicity Criteria (CTC) (10, 25) were recorded. The patients were followed up for 3 months, 1, 2 and 3 years in our institution after discharge, and follow-up was carried out by telephone or E-mail thereafter. The follow-up was completed and the postoperative long-term KPS was subjectively conducted by three experienced neurosurgeons (**Supplementary Figure 2**). The median follow-up of the present cohorts was 68 months with an interquartile range (IQR): 41 to 104 months.

### Statistical Analysis

#### Evaluation of Function Outcome

Kruskal–Wallis H test was calculated in the population distribution rate of each KPS before surgery and 3 years after surgery. The differences between preoperative KPS and postoperative long-term KPS were depicted based on each level of preoperative KPS. We further described the population distribution frequency of postoperative long-term function conversion using composite column-diagram and heat map. The remaining symptoms related to postoperative long-term function outcomes were shown by proportion and displayed in different levels of KPS (100–80, 70–50 and ≤40). Spearman correlations between symptoms and KPS were performed to determine a pivotal symptoms’ cluster affecting postoperative long-term independence (R ≥ | ± 0.5| means significantly strong correlation). Furthermore, the Kaplan–Meier curves regarding recurrence related to postoperative long-term independence were illustrated with the log-rank test.

#### Comparison of Clinical Features

Demographic and clinical data were generalized by mean ± SD or median and IQR for continuous variables and counts with proportions for categorical features. Association between function outcomes (postoperative long-term independence or non-independence) and variables of interest was tested with a Chi-square, Fisher’s exact test or Mann–Whitney U test, as necessary. A correlated matrix was used to estimate all explanatory variables for collinearity, and plausible interaction terms were tested with a variance inflation factor (VIF). The multivariate analysis was performed by Logistic regression model. Potential-risk estimators with *P* value <0.05 in the univariate Logistic regression analysis or based on clinical importance, scientific knowledge and predictors identified in previously published articles (7, 10, 26–29), among all variables, were tested in the multivariate model. Backward stepwise selection with the Akaike information criterion (AIC) was used to identify variables for the multivariable Logistic regression model and Odd ratios (ORs) were presented with their 95% confidence intervals (CIs).

#### Model Development and Performance Evaluation

To conduct early postoperative consultation, creating a simple-to-use function evaluation score that could be utilized by clinicians to predict the possibilities of postoperative long-term non-independence in elderly meningioma remains of importance. We generated a nomogram using each weighted covariate derived from the fitted multivariate Logistic regression model except for the variable of recurrence during follow-up due to its unpredictability. The C-index was graphically illustrated to compute the area under the curve (AUC) for the model, and Hosmer–Lemeshow goodness-of-fit was performed. The calibration of the model was performed graphically, which as the main approach was implemented to evaluate consistency between the predicted probability values and actual probability values. And scattered points on the 45-degree diagonal reference line of the graphical indicated stable calibration. Statistical analysis and nomogram construction and validation were performed by R v3.6.3. All tests were two-sided with a statistically significant *P* value <0.05.

## Results

### Patient Features

The clinical histories of 549 elderly individuals admitted to our institution between 2010 and 2016 were reviewed. A total of 470 patients met the inclusion criteria. **Supplementary Figure 1** shows ineligible patients’ details. There were 138 males and 332 females (ratio: 1:2.4) with a mean age of 68.79 years (range: 65–79). There were 175 (37.2%) patients with postoperative long-term KPS ≤70, 32 (6.8%) patients with recurrence, and 29 (6.0%) with mortality during follow-up. Additionally, recurrence presented in follow-up commonly combined with long-term KPS ≤70 (*P* < 0.001) (**Supplementary Figure 3**). Baseline details were summarized in **Supplementary Table 4**.

### Postoperative Long-Term Independence

**Figure 1:**
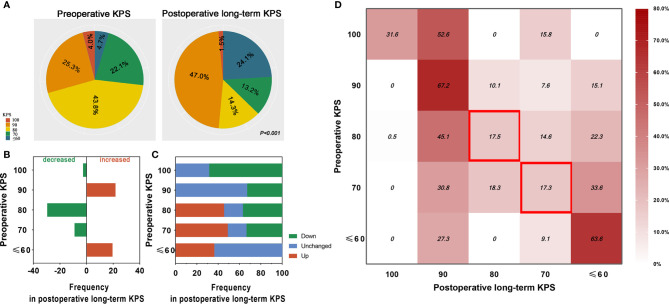
Comparison and distribution of elderly meningioma population between preoperative KPS and postoperative long-term KPS. **(A)** Distribution of elderly meningioma patients before and 3 years after surgery in different levels of KPS (P < 0.001). The red, orange, yellow, green, and blue colors represent KPS 100, 90, 80, 70 and ≤ 60, respectively. **(B)** Difference of elderly meningioma patients’ distribution before and 3 years after surgery in different gradients of preoperative KPS. The green and red columns represent decreased and increased rate of the elderly population. **(C)** Ability to the conversion of postoperative long-term KPS in elderly with meningiomas. Red, blue, and green columns represent the rate of better, unchanged, and worse conversion, respectively. **(D)** Distribution of elderly meningioma population of each postoperative long-term KPS in different gradients of preoperative KPS. Contents in the red blank box emphasize only less than 20% of elderly patients with preoperative KPS 80 or 70 maintain unchanged KPS 3 years after resection.

Compared with preoperative values, the human distribution of postoperative long-term KPS were significantly different, with 1.5 *vs.* 4.0%, 47.0 *vs.* 25.3%, 14.3 *vs.* 43.8, 13.2 *vs.* 22.1%, and 24.1 *vs.* 4.7%, respectively (H = 13.550, *P* < 0.001) (**Figure 1A**). Furthermore, our results showed that the proportion of KPS 90 and ≤60 increased 21.7 and 19.4% after 3 years, respectively, and the proportion of KPS 80 and 70 decreased 29.5 and 8.9% after 3 years, respectively (**Figure 1B**). To explore the postoperative long-term conversion of each KPS (**Figure 1C**) and transform-based distribution (**Figure 1D**), we found that 31.6% of patients with preoperative KPS 100 kept the same KPS after 3 years, and the KPS of all the remaining patients had decreased, but 52.6% of patients only slightly decreased to KPS 90 (**Figure 1D**). Among patients with preoperative KPS 90 and ≤60, 67.2 and 63.6% stay with unchanged KPS after 3 years, and all the remaining declined and improved to the extent across the board, respectively (**Figures 1C, D** **)**. Most notably, we found that in patients with preoperative KPS 80, only 17.5% of the elderly maintained the same KPS 3 years after surgery, and the remaining emerged with distinct polarization trend that 45.6% of patients experienced better postoperative long-term independence while 36.9% as worse (**Figures 1C, D** **)**. A similar phenomenon that only 17.3% of the elderly preserved the KPS unchanged 3 years after surgery, but 82.7% of elderly individuals experienced polarization trend in postoperative long-term independence recovery (49.1% patients experienced better independence while 33.6% as worse) was investigated when elderly patients harbored preoperative KPS 70 (**Figures 1C, D** **)**.

### Postoperative Long-Term Remaining Symptoms

**Figure 2:**
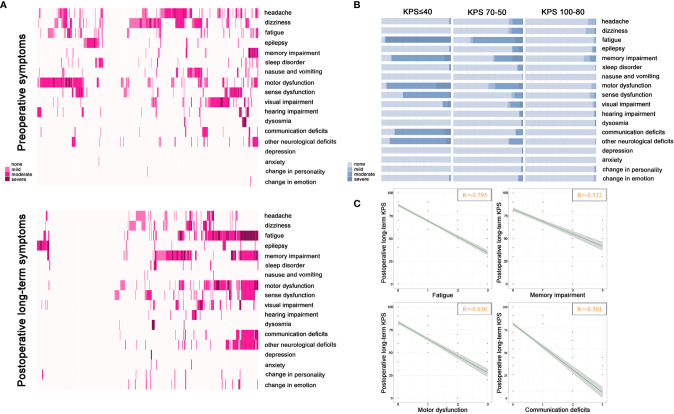
Distribution of symptoms and correlation with KPS. **(A)** The description of preoperative symptoms and postoperative long-term remaining symptoms. The color of the block from light pink to dark pink represents none, mild, moderate, and severe. **(B)** Correlation between severity and quantity of each symptom and different levels of postoperative long-term KPS. The color from light blue to dark blue represents none to severe. **(C)** Correlations between severity of remaining symptoms and postoperative long-term KPS. Significantly correlated symptoms include fatigue (R = −0.795), memory impairment (R = −0.512), motor dysfunction (R = −0.636) and communication deficit (R = −0.501) (*P* < 0.001).

To evaluate the correlation between remaining symptoms and long-term independence after surgery, **Figure 2A** showed 28.5% of elderly patients were recorded as asymptomatic status in a follow-up but 71.5% with symptoms where the top three of the highest frequent deficits were fatigue (32.3%), memory impairment (30.6%), and motor dysfunction (26.4%). In terms of severity of symptoms, the majority of symptoms were experienced as moderate or severe and less often as mild when elderly patients harbored postoperative long-term KPS 70–50 or ≤40 (**Figure 2B**). We further found that the quantity of symptoms was significantly increased following the attenuated long-term KPS after surgical procedure (**Figure 2B**). Additionally, a graphical representation of the Spearman correlations between remaining symptoms and postoperative long-term KPS was presented in **Figure 2C**. One pivotal symptomatic cluster including fatigue (R = −0.795), memory impairment (R = −0.512), motor dysfunction (R = −0.636) and communication deficits (R = −0.501) was confirmed as adverse events significantly associated with attenuated long-term independence.

### Comparison of Clinical Characteristics Between Postoperative Long-Term Independent and Non-Independent Cohorts

The proportions of patients with advanced age at surgery, recurrent meningioma, presenting symptoms, attenuated preoperative KPS, preoperative comorbidities, tumor located in the skull base, tumor maximal diameter > 6cm, irregular tumor shape, tumor involved nerves, medical/surgical complications, high risk of WHO grade and biological behavior, attenuated KPS on discharge, radiotherapy after surgery and recurrence during follow-up were higher in the postoperative long-term non-independent cohort (*P* < 0.05), while that of those with GTR was lower (*P* < 0.05) (**Table 1** **)**. However, the frequency of tumor in the right or left hemisphere and single or multiple lesions was found to be comparable between the two cohorts. Furthermore, we found a tendency that the presence of tumor involved motor cortex was prevalent in postoperative long-term non-independent cohorts, but this difference was not statistically significant (*P* = 0.084).

**Table 1:** Univariate comparison between both cohorts.

	Long-term independent status N (%)		*P* value
	Yes N = 295(62.8)	No N = 175(37.2)	
**Gender N(%)**			
Female	217(73.6)	115(65.7)	0.071
Male	78(26.4)	60(34.3)	
**Age at surgery, years**			0.000*
65–69	218(73.9)	98(56.0)	
70–74	59(20.0)	54(30.9)	
≥75	18(6.1)	23(13.1)	
**Presenting symptoms^a^ N(%)**			0.001*
Yes	255(86.4)	168(96.0)	
No	40(13.6)	7(4.0)	
**Preoperative comorbidities N(%)**			0.043*
Yes	152(51.5)	107(61.1)	
No	143(48.5)	68(38.9)	
**Preoperative *γ*-knife N(%)**			0.500
Yes	7(2.4)	6(3.4)	
No	288(97.6)	169(96.6)	
**Recurrent meningioma N(%)**			0.000***
Yes	9(3.1)	38(21.7)	
No	286(96.9)	137(78.3)	
**Other surgery history N(%)**			0.916
Yes	111(37.6)	65(37.1)	
No	184(62.4)	110(62.9)	
**Smoking N(%)**			0.584
Yes	29(9.8)	20(11.4)	
No	266(90.2)	155(88.6)	
**Drinking N(%)**			0.430
Yes	18(6.1)	14(8.0)	
No	277(93.9)	161(92.0)	
**Tumor side N(%)**			0.080
Left/Right side	257(87.1)	142(81.1)	
midline	38(12.9)	33(18.9)	
**Multiple lesions N(%)**			0.847
Yes	14(4.7)	9(5.1)	
No	281(95.3)	166(94.9)	
**Location N(%)**			0.015*
convexity	91(30.8)	33(18.9)	
Falx/sagittal sinus	61(20.7)	47(26.9)	
Tentorium	32(10.8)	12(6.9)	
Skull base	106(35.9)	80(45.7)	
intraventricular	5(1.7)	3(1.7)	
**Motor cortex involved N(%)**			0.084
Yes	44(14.9)	37(21.1)	
No	251(85.1)	138(78.9)	
**Maximal diameter, cm**			0.000***
≤6	277(93.9)	143(81.7)	
>6	18(6.1)	32(18.3)	
**Tumor shape N(%)**			0.005*
Regular	257(87.1)	135(77.1)	
Irregular	38(12.9)	40(22.9)	
**Nerves involved N(%)**			0.003*
Yes	30(10.2)	35(20.0)	
No	265(89.8)	140(80.0)	
**WHO grade and biological behavior N(%)**			0.000***
Low risk of recurrence and aggressive behavior	269(91.2)	122(69.7)	
High risk of recurrence and aggressive behavior	26(8.8)	53(30.3)	
**Resection extent N(%)**			0.000***
GTR	269(91.2)	136(77.7)	
STR	26(8.8)	39(22.3)	
**Any medical/surgical complications N(%)**			0.007*
Yes	60(20.3)	55(31.4)	
No	235(79.7)	120(68.6)	
**Radiotherapy after surgery N(%)**			0.004*
Yes	7(2.4)	14(8.0)	
No	288(97.6)	161(92.0)	
**Long-term antiepileptic drug therapy**			0.142
Yes	10(3.4)	11(6.3)	
No	285(96.6)	164(93.7)	
**Recurrence during follow-up**			0.000***
Yes	5(1.7)	27(15.4)	
No	290(98.3)	148(84.6)	
**Preoperative KPS median (IQR)**	80(80,90)	80(70,80)	0.000***
**KPS on discharge median (IQR)**	90(80,90)	80(70,90)	0.000***
**Postoperative long-term KPS median (IQR)**	90(90,90)	60(50,70)	0.000***

^a^Presenting symptoms refer to **Supplementary Table 3**.

*P < 0.05;***P < 0.001.

### Univariate and Multivariate Logistic Regression Analysis in Postoperative Long-Term Non-Independence

We selected 15 candidates fitting both the clinical and statistical criteria of *P <*0.05 into a multivariate Logistic regression analysis. These candidate predictors included older age at surgery, presenting symptoms, attenuated preoperative KPS, preoperative comorbidities, recurrent meningioma, different tumor locations, tumor maximal diameter >6cm, nerves involved, irregular tumor shape, high risk of WHO grade and biological behavior, STR, medical/surgical complications, attenuated KPS on discharge, radiotherapy after surgery and recurrence during follow-up (**Table 2**).

**Table 2:** Logistic regression showing the association of variables with postoperative long-term non-independent status.

	Univariable	*P* value	Multivariable	*P* value
	OR(95%CI)		OR(95%CI)	
**Gender (male/female)**	1.452(0.968–2.177)	0.072		
**Age at surgery, years**				
65–69	1	0.000***	1	0.000***
70–74	2.036(1.312–3.159)	0.002*	2.631(1.545–4.481)	0.000***
≥75	2.842(1.467–5.506)	0.002*	3.833(1.667–8.812)	0.002*
**Presenting symptoms (yes/no)**	3.765(1.648–8.602)	0.002*	1.865(0.667–5.214)	0.235
**Preoperative comorbidities (yes/no)**	1.480(1.012–2.165)	0.043*	1.405(0.877–2.250)	0.157
**Preoperative γ-knife (yes/no)**	1.461(0.483–4.418)	0.502		
**Recurrent meningioma (yes/no)**	8.814(4.145–18.745)	0.000***	7.791(3.202–18.954)	0.000***
**Other surgery history (yes/no)**	0.980(0.665–1.442)	0.916		
**Smoking (yes/no)**	1.184(0.648–2.163)	0.584		
**Drinking (yes/no)**	1.338(0.648–2.763)	0.431		
**Tumor side (left or right side/midline)**	0.636(0.382–1.059)	0.082		
**Multiple lesions (yes/no)**	1.088(0.461–2.569)	0.847		
**Location**				
Convexity	1	0.016*	1	0.022*
Falx/sagittal sinus	2.125(1.225–3.685)	0.007*	2.346(1.179–4.667)	0.015*
Tentorium	1.034(0.477–2.242)	0.932	1.455(0.564–3.754)	0.437
Skull base	2.081(1.271–3.407)	0.004*	2.683(1.383–5.205)	0.004*
Intraventricular	1.655(0.374–7.310)	0.507	4.465(0.836–23.857)	0.080
**Motor cortex involved (yes/no)**	1.529(0.943–2.482)	0.085		
**Maximal diameter, cm (>6/≤6)**	3.444(1.868–6.349)	0.000***	3.089(1.471–6.488)	0.003*
**Tumor shape (irregular/regular)**	2.004(1.227–3.272)	0.005*	1.387(0.728–2.646)	0.319
**Nerves involved (yes/no)**	2.208(1.301–3.748)	0.003*	3.144(1.585–6.235)	0.001*
**WHO grade and biological behavior (High risk/Low risk)**	4.495(2.684–7.528)	0.000***	2.294(1.193–4.408)	0.013*
**Resection extent (STR/GTR)**	2.967(1.734–5.078)	0.000***	1.477(0.763–2.858)	0.247
**Any medical/surgical complications (yes/no)**	1.795(1.171–2.751)	0.007*	1.568(0.911–2.698)	0.105
**Radiotherapy after surgery (yes/no)**	3.578(1.415–9.045)	0.007*	0.503(0.158–1.603)	0.245
**Long-term antiepileptic drug therapy (yes/no)**	1.912(0.795–4.598)	0.148		
**Recurrence during follow-up (yes/no)**	10.581(3.993–28.040)	0.000***	10.296(3.253–32.585)	0.000***
**Preoperative KPS**	0.937(0.916–0.958)	0.000***	0.964(0.938–0.991)	0.009*
**KPS on discharge**	0.961(0.945–0.977)	0.000***	0.967(0.951–0.984)	0.000***

*P < 0.05; ***P < 0.001.

Subsequent multivariate analysis of variables indicated that older age (70–74 *vs* 65–69 OR: 2.631; 95% CI: 1.545–4.481 and ≥75 *vs* 65–69 OR: 3.833; 95% CI: 1.667–8.812), recurrent meningioma (OR: 7.791; 95% CI: 3.202–18.954), tumor location in the falx/sagittal sinus (OR:2.346; 95% CI:1.179–4.667), tentorium (OR:1.455; 95% CI: 0.564–3.754), skull base (OR: 2.683; 95% CI: 1.383–5.205) and intraventricular (OR: 4.465; 95% CI: 0.836–23.857), tumor maximal diameter >6 cm (OR: 3.089; 95% CI: 1.471–6.488), nerves involved (OR: 3.144; 95% CI: 1.585–6.235), high risk of WHO grade and biological behavior (OR: 2.294; 95% CI: 1.193–4.408), lower preoperative KPS (OR: 0.964; 95% CI: 0.938–0.991), lower KPS on discharge (OR: 0.967; 95% CI: 0.951–0.984) and recurrence during follow-up (OR: 10.296; 95% CI: 3.253–32.585) were independent risk factors for postoperative long-term non-independence following elderly meningioma resection (**Table 2**).

### Individualized Prediction Nomogram and Model Performance

**Figure 3:**
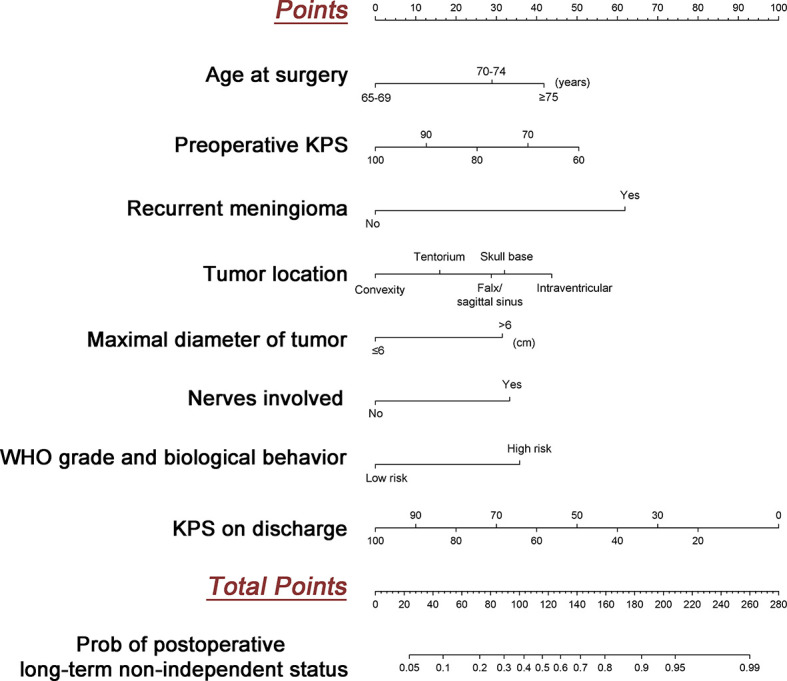
Development of the predicted model. The nomogram to predict postoperative long-term non-independent status in the elderly with meningioma is created based on eight risk factors incorporated into multivariate logistic regression. For instance, a patient with age of 70, preoperative KPS at 80, primary tumor, maximal diameter at 4 cm, location in the skull base, nerves involved, low risk WHO grade and biological behavior, and KPS on discharge at 80 would have a total of 140.5 points (29 points for age at 70, 25 points for preoperative KPS at 80, 0 point for the primary tumor, 0 point for maximal diameter at 4cm, 32.5 points for location in the skull base, 34 points for nerves involved, 0 points for low risk WHO grade and biological behavior, and 20 points for KPS on discharge at 80), for a predicted postoperative long term non-independent status of 68%.

**Figure 4:**
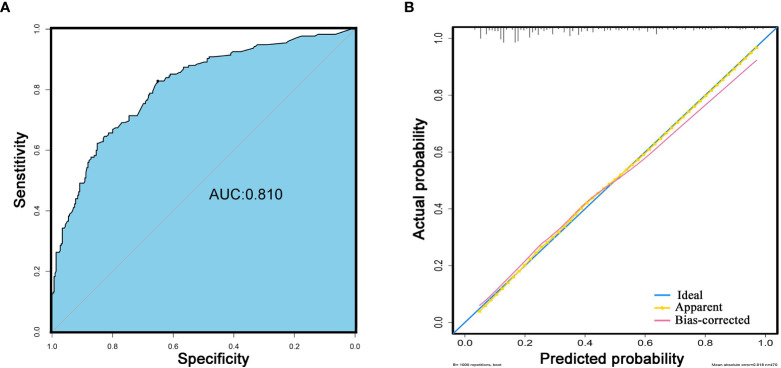
Performance of the model. **(A)** The discriminative ability for the prediction of postoperative long-term non-independent status in the elderly with meningioma. Blue background represents the area of AUC (C-index: 0.810). **(B)** Calibration curves of model. The blue line represents the ideal fit. The yellow line represents apparent model-predicted probabilities and the pink line represents bias-corrected estimates with 1000-fold bootstrapping.

An easy-to-use scoring assessment to predict postoperative long-term functional outcomes remains of importance when elderly patients underwent discharge. Therefore, established risk predictors besides the variable of recurrence during follow-up incorporated into multivariate Logistic regression analysis were selected to configure a nomogram (**Figure 3**) (**Supplementary Table 5**). The discriminative ability of the model using the C-index was 0.810 (**Figure 4A**) without collinearity (VIF = 1.052–1.175). **Figure 4B** displays the calibration plot of the model, indicating a good fit between observed and predicted values. The Hosmer–Lemeshow goodness-of-fit test yielded a non-significant statistic (χ2 = 6.081, *P* = 0.638), which suggested that there was no departure from the perfect fit.

## Discussion

Meningiomas are the common primary intracranial tumor, and the majority are benign and can be surgically cured through complete tumor resection (2). With an aging global population, the incidence of meningiomas in patients aged 65 years and older has also steadily increased (1). Given the increased risk of adverse outcomes among older patients after a surgical procedure and oncologic curability, measures of treatment success have appropriately shifted to more patient-centered metrics, including postoperative long-term maintenance of independence in meningioma patients. Our study demonstrated the postoperative long-term independence status varied considerably across meningioma patients, especially patients with preoperative KPS equal to 80 and 70 have obvious polarization trends in postoperative long-term independence. Then, similar to what has been reported in a previous study about glioma (30), we have found that fatigue, memory impairment, motor dysfunction, and communication deficits were consistently highly correlated with long-term loss of independence in elderly meningioma patients. Finally, we identified several independent risk factors to develop and internally validate a prediction model for postoperative long-term loss of independence in the elderly with meningioma.

So far, this is the largest reported study of postoperative long-term independence in elderly meningioma patients. In this study, we report the long-term independent status of 470 meningioma patients treated with surgical resection. The KPS is a validated and widely used function evaluation scale to assess performance status in clinical practice region (31). In several recent studies, functional independence is defined by a KPS >70 (17, 22). Assessing evolution over of the KPS is important because functional independence is a key factor for QoL and is emerging as an important endpoint in clinical trials (13–15, 32). Although the HRQoL scale could obtain multi-dimensional details of function deficits, the complex assessment process and high training demand might limit the use of both clinicians and patients (3, 33). Therefore, in the present study, we used the KPS score to evaluate postoperative long-term independence status in elderly patients with meningioma. Our results demonstrated that almost half of elderly meningioma patients have improved long-term independent status after surgical resection of tumors, while a proportion of patients might have worse non-independent status. This is the first time that we considered postoperative long-term independence in elderly meningioma patients that comprehensively presents a polarization trend. Through further analyzing the change of KPS scores before and after surgery, we found that there were slightly decreased postoperative long-term KPS scores in more than half of patients with perfect preoperative independent status (KPS scores of 100), but the degree of decline had little impact on independence. Whereas, the majority of elderly meningioma patients with preoperative KPS of 90 or ≤60 would keep the same independent status even undergoing surgery. This could mean that in elderly meningioma patients with better or worse preoperative functional status, tumor resection would provide very little space for improvement or deterioration of function. Most notably, though, among elderly meningioma patients with preoperative KPS of 70 or 80, only less than 20% of patients preserved the original independence status, while the remaining patients showed obvious polarization trend and were evenly split between improvement and deterioration of postoperative long-term independence. This finding is a little bit different from previous studies that reported worse prognostic outcomes after resection in meningioma were closely associated with preoperative KPS less than 70 (26, 34). The heterogeneity of aging physiology and surgical attack may be an underlying cause of our findings. Therefore, for elderly meningioma patients with preoperative KPS of 70 or 80, a comprehensive strategy involving screening for predisposing factors of postoperative non-independence and early prevention of modifiable factors should be established in this population.

In order to preserve and improve the postoperative long-term independence in elderly meningioma patients, we further explored the risk factors to facilitate early recognition of high-risk population and early prevention of modifiable factors. Until now, few studies have investigated independent risk factors of postoperative long-term QoL, also including long-term independence in meningioma patients. Unsurprisingly, several independent risk factors that have previously been associated with functional deficits, recurrence, and death in meningioma patients, including poor performance before operation and on discharge, advanced age, large tumor diameter and high risk of grade and biological behavior were also found to increase the elderly meningioma patients’ susceptibilities to long-term non-independent status (9, 10, 26, 27, 29). In addition, we also confirmed that tumor located in the skull base was an independent risk factor for long-term non-independent status in our population. Previous studies suggested that brain tumors located in the cerebellopontine angle and anterior clinoidal might affect postoperative functional status and QoL, perhaps because they were always adjacent to important nerves and vessels (26). Besides, Hischam Bassiouni et al. reported that approximately one-third of patients with meningioma involved visual nerve could achieve improved functional outcomes after excision, but the majority remained unchangeable and even worsened (35). We found a similar phenomenon that a close correlation between postoperative long-term non-independent status and tumor involved nerves in our cohort. Furthermore, our results showed that recurrent tumor before surgery and tumor recurrence in follow-up were the most significant risk factors for postoperative long-term non-independence with the top two highest weight in the present study. Recurrence of meningioma not only brings a second attack to the brain but could also mean more malignant biological behavior. Therefore, although meningiomas are mostly benign tumors, postoperative follow-up cannot be ignored. Some variables including presenting symptoms, preoperative comorbidities, tumor shape, extent of resection, medical/surgical complications and radiotherapy after surgery in our study had high significance in univariate analysis but were not selected into the final multivariable analysis, which means there may be some indirect association between these variables and independent predictors. More research studies are still needed for these variables. Finally, in order to identify high-risk patients for postoperative long-term non-independence and early implementation of proactive multifactorial interventions, we developed and internally validated a prediction model for postoperative long-term non-independence in elderly meningioma patients. We need to draw up the early systemic rehabilitation plan for the high-risk population on discharge, so we did not incorporate the factor of recurrence during follow-up into our model. Our prediction model consists of eight risk factors that are readily available on discharge and has a high predictive value. However, the optimal cut-off point of the model and the most effective preventive interventions for our population need to be studied shortly.

Our study also has several limitations. First, this was a single-center study and the model developed was only internally validated and not externally verified. Therefore, our finding might be limited for widely generalizing in other regions and races. Second, due to the retrospective nature of this project, not all information, mainly including long-term independence trajectory for older adults with meningiomas was available in our study, and some certain biases should be generated inevitably. Third, collection and record of symptoms were referred to QLQ-BN20 questionnaires. Owing to some common symptoms in patients with brain tumors, such as cognitive decline, emotion deficits and hearing impairments were not included in QLQ-BN20 questionnaires (3). We added some extra symptoms by patients’ complaints and psychiatric diagnosis. While, some symptoms in QLQ-BN20, such as diarrhea or appetite loss, were not reported in our study because few presented during the follow-up. Therefore, a specific meningioma symptom assessment may be urgently needed in future studies. Fourth, HRQoL scales are widely used in the assessment of QoL of patients with tumors because they could obtain multi-dimensional details of functional deficits. Functional independence is a key factor for QoL and also the primary outcome in the present study. Therefore we used KPS as an assessment tool of independent status. Several previous studies reported the close association between KPS and HRQoL or cognition (16, 32, 33) but KPS might not be sensitive enough to cognition impairment (20). This is also the most important limitation in our study.

## Conclusions

In this study, maintenance of postoperative long-term independence appeared to be a polarization trend, especially in patients with preoperative KPS equal to 80 and 70. The remaining symptom clusters including fatigue, memory impairment, motor dysfunction and communication deficits were highly correlated with non-independence 3 years after surgery, and strategies targeting these domains from an early point in treatment may offer the optimal approach for maximizing postoperative long-term independence in these population. Furthermore, identifying the potential risk predictors and developing the prediction model might also help to draw up the early rehabilitation plan for patients with high risk of long-term loss of independence.

## Data Availability Statement

The raw data supporting the conclusions of this article will be made available by the authors, without undue reservation.

## Ethics Statement

The studies involving human participants were reviewed and approved by the Ethics Committee of Beijing Tiantan Hospital. The patients/participants provided their written informed consent to participate in this study.

## Author Contributions

GZ, ZJ, and HL conceptualized and designed the study. HL, HH, XZ, XR, YC, and DS collected the data. GZ, HL, and HH analyzed and interpreted the data. HL and HH drafted the article. GZ, ZJ, SL, YW, HL, HH, and XZ critically revised the article. GZ, ZJ, and SL supervised the study. All authors contributed to the article and approved the submitted version.

## Funding

This work was supported by the Beijing Outstanding Talent Training Foundation, Youth Backbone Individual Project (2018000021469G230), National Natural Science Foundation of China (81801042), and Beijing Hospital Authority Youth Programme (20190504). The funds had no role in the study design, data collection and analysis, decision to publish, or manuscript preparation.

## Conflict of Interest

The authors declare that the research was conducted in the absence of any commercial or financial relationships that could be construed as a potential conflict of interest.

## Supplementary Material

Supplementary Figure 1 Consort chart showing the selection path for patients to be included this study.

Supplementary Figure 2 Consort chart showing the process of follow-up.

Supplementary Figure 3 Kaplan–Meier curves of recurrence after surgery for the elderly who underwent long-term independence and non-independence after meningioma resection.
