# Supplementary figures and images for: Postoperative Long-Term Independence Among the Elderly With Meningiomas: Function Evolution, Determinant Identification, and Prediction Model Development
Source: Front Oncol. 2021 Mar 5;11:639259. doi: 10.3389/fonc.2021.639259 (PMC7982808; doi:10.3389/fonc.2021.639259)

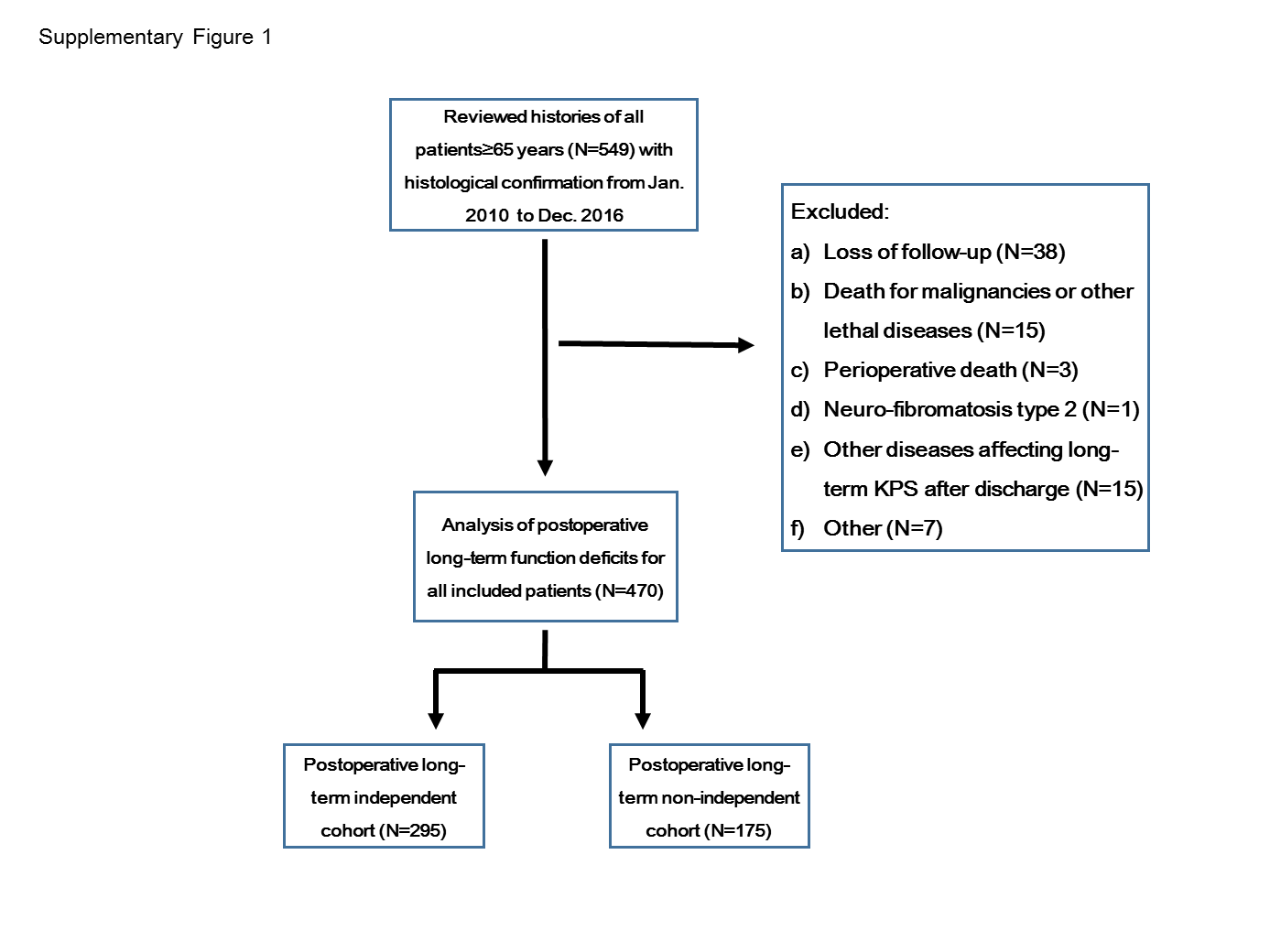

Supplement: Supplementary Figure 1 — Consort chart showing the selection path for patients to be included this study. [file Image_1.tif]

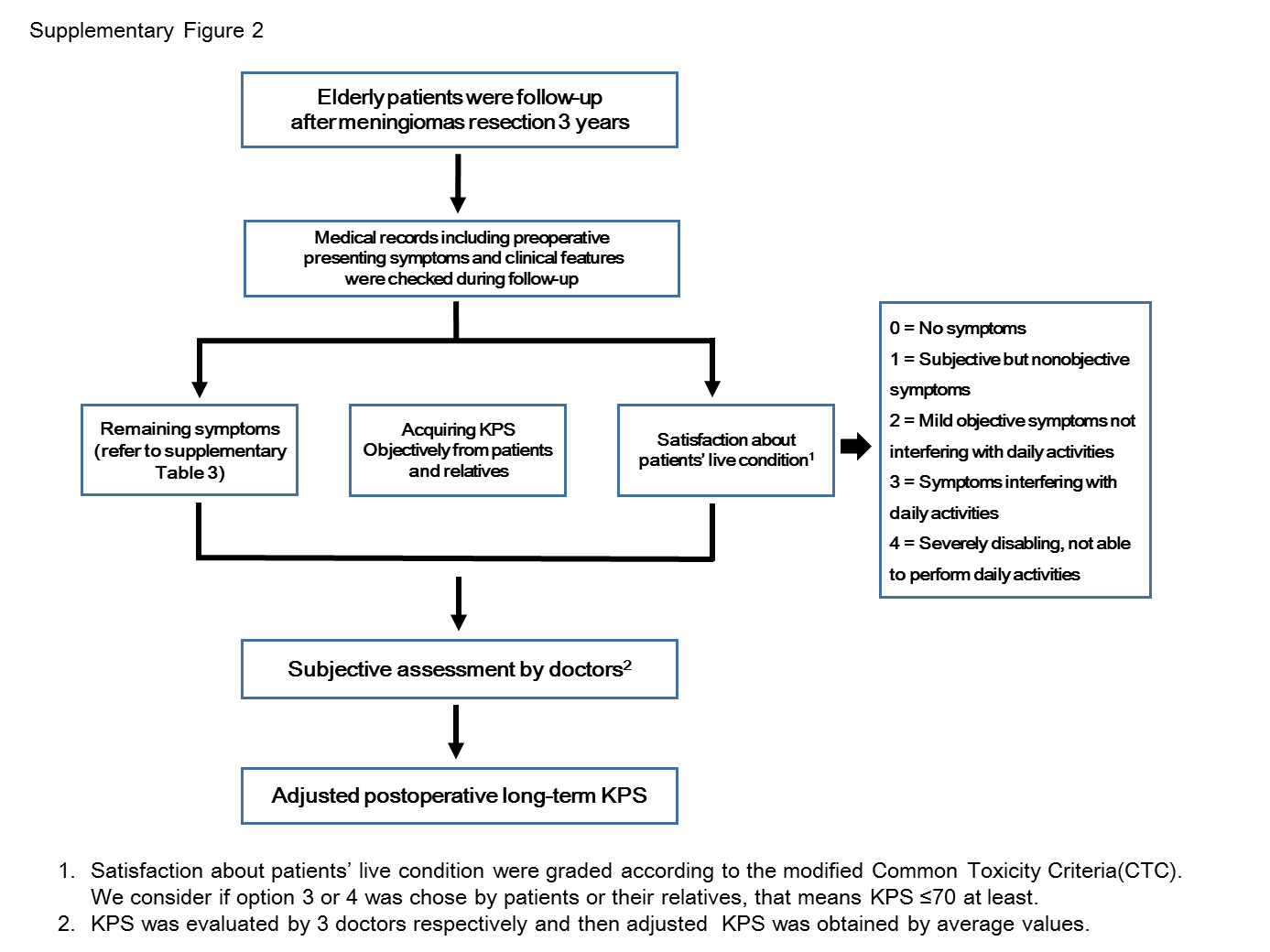

Supplement: Supplementary Figure 2 — Consort chart showing the process of follow-up. [file Image_2.tif]

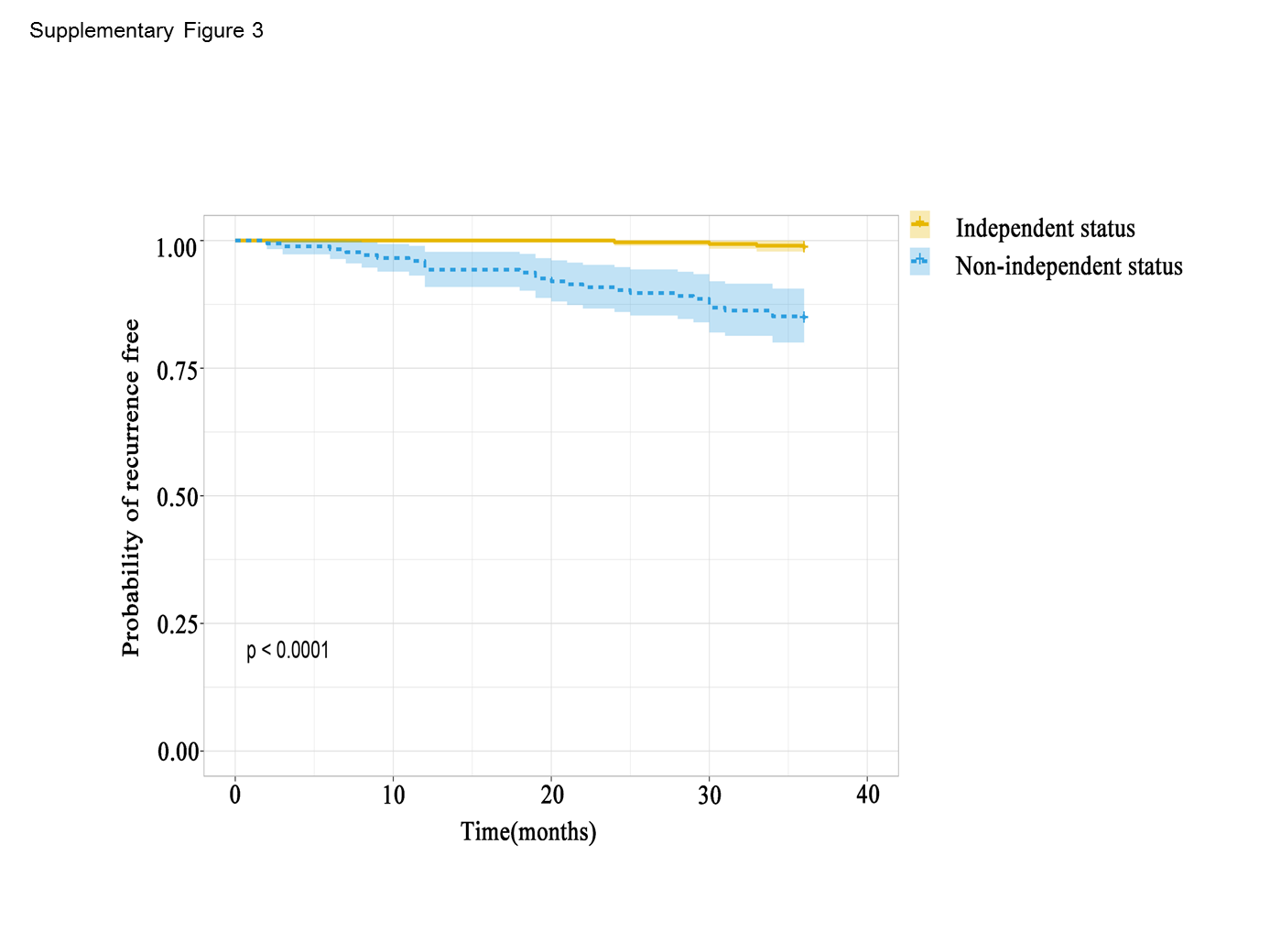

Supplement: Supplementary Figure 3 — Kaplan–Meier curves of recurrence after surgery for the elderly who underwent long-term independence and non-independence after meningioma resection. [file Image_3.tif]
