# Supplementary material for: Postoperative Long-Term Independence Among the Elderly With Meningiomas: Function Evolution, Determinant Identification, and Prediction Model Development
Source: Front Oncol. 2021 Mar 5;11:639259. doi: 10.3389/fonc.2021.639259 (PMC7982808; doi:10.3389/fonc.2021.639259)
Supplement: Supplementary file 4 [file DataSheet_1.docx]

| **Supplementary Table 1**  **Predictors initially considered** | |
| --- | --- |
| **Factors** | **Description** |
| **Pre-operation** | **Patient characteristics:**   1. Gender(male/female) 2. Age at surgery( categorized as 65-69 ,70-74 & ≥75 years ) 3. recurrent meningioma ( categorized as Yes & No) 4. Other surgery history(categorized as Yes & No) 5. Preoperative γ -knife (categorized as Yes & No) 6. Smoking (categorized as Yes & No) 7. Drinking (categorized as Yes & No) 8. Presenting symptoms (categorized as Yes & No) 9. Preoperative KPS ( continuous variable )   **Preoperative comorbidities:**   1. Comorbidities (hypertension, diabetes mellitus, coronary artery disease, cerebrovascular accident, pulmonary disease and hydrocephalus, categorized as Yes & No)   **Radiology features:**   1. Tumor location(categorized as convexity, Falx/sagittal sinus Tentorium, intraventricular, skull base) 2. Tumor side (categorized as Midline & Left/right) 3. Single or multiple lesions (categorized as Yes & No) 4. Maximal diameter (from the edge of the tumor to the edge of the adjacent edema, categorized as ≤6 and>6cm) 5. Motor cortex involved (categorized as Yes & No) 6. Tumor shape(categorized as Regular & Irregular) |
| **Intra-operation** | 1. tumor involved nerves (categorized as Yes & No ) |
| **Post-operation** | **Perioperative management:**   1. Pathological report( categorized as low risk of recurrence and aggressive behavior & high risk of recurrence and aggressive behavior ) 2. Operative report( categorized as gross total resection & subtotal resection) 3. KPS on discharge ( continuous variable )   **Perioperative complication:** (categorized as Yes & No)   1. CNS infection 2. Wound infection 3. Intracranial hemorrhage 4. Subdural hematoma 5. Cerebrovascular accident 6. Hydrocephalus 7. Reoperation(recraniotomy/shunt/drainage) |
| **Long-term follow-up**  **(discharge after 3 years)** | Recurrence during follow-up(categorized as Yes & No)  Radiotherapy after surgery(categorized as Yes & No)  Long-term antiepileptic drugs therapy (categorized as Yes & No)  Death during follow-up  Long-term remaining symptoms  Long-term KPS ( categorized as＞70 or ≤70) |

**Supplementary Table 2**

**2016 WHO classification of Tumors of the Central Nervous System ---- Meningioma**

| **Meningiomas with low risk of recurrence and aggressive behavior** | | |
| --- | --- | --- |
| **Pathological Type** | **WHO grade** | **ICD-0 code** |
| **Meningothelial** | I | 9531/0 |
| **Fibrous(fibroblastic)** | I | 9532/0 |
| **Transitional(mixed)** | I | 9537/0 |
| **Psammomatous** | I | 9533/0 |
| **Angiomatous** | I | 9534/0 |
| **Microcystic** | I | 9530/0 |
| **Secretory** | I | 9530/0 |
| **Lymphoplasmacyte-rich** | I | 9530/0 |
| **Metaplastic** | II | 9530/0 |
| **Meningiomas with high risk of recurrence and aggressive behavior** | | |
| **Chordoid** | II | 9538/1 |
| **Clear cell** | II | 9538/1 |
| **Atypical** | II | 9539/1 |
| **Papillary** | III | 9538/3 |
| **Rhabdoid** | III | 9538/3 |

**Supplementary Table 3**

**Function-related symptoms ^a^**

| **Neurological deficits** | Headache, dizziness, fatigue, epilepsy, memory impairment, sleep disorder (drowsiness, insomnia), nasusea and vomiting, motor dysfunction (motor deficit, weakness, gait instability), sense dysfunction (sense deficit, pain, numbness, paresthesia), visual impairment, Hearing impairment, dysosmia, communication dificits, other neurological deficit. |
| --- | --- |
| **Other deficits ^b^** | Depression, anxiety, change in personality, change in emotion |

^a^ All symptoms were presented based on European Organization for Research and Treatment of Cancer Quality of Life Questionnaire-Brain Neoplasm 20 (QLQ-BN20) which was modified appropriately due to the existing limited items and the characteristics of elderly patients.

^b^ Other deficits reported related mental symptoms which were verified by psychiatric diagnosis records.

**Supplementary Table 4**

**Clinical features of elderly individuals with meningiomas**

| **Gender N(ratio)** |  |
| --- | --- |
| Male/Female | 138/332(1/2.4) |
| **Age at surgery, years** |  |
| Mean±SD | 68.79±3.484 |
| Range | 65-79 |
| **Preoperative symptoms N(%)** | 423(87.0) |
| **Location N(%)** |  |
| convexity | 124(26.4) |
| Falx/parasagittal | 108(23.0) |
| tentorial | 44(9.4) |
| Skull base | 186(39.6) |
| intraventricular | 8(1.7) |
| **WHO grade and biological behavior N(%)** |  |
| Low risk of recurrence and aggressive behavior | 391(83.2) |
| High risk of recurrence and aggressive behavior | 79(16.8) |
| **Tumor maximal diameter, cm** |  |
| Mean±SD | 4.501±1.5553 |
| **Resection extent N(%)** |  |
| GTR | 405(86.2) |
| STR | 65(13.4) |
| **Preoperative KPS (IQR)** | (70,80) |
| **KPS on discharge (IQR)** | (80,90) |
| **Postoperative Long term KPS N(%)** |  |
| >70 | 295(62.8) |
| ≤70 | 175(37.2) |
| **Recurrence during follow-up** | 32(6.8) |
| **Death during follow-up N(%)** | 29(6.0) |

**Supplementary Table 5**

**Model development associated with postoperative long-term non-independence**

|  | **Model** | | |  |
| --- | --- | --- | --- | --- |
|  | **OR (95%CI)** | | | ***P* value** |
| **Age at surgery, years** |  | | |  |
| 65-69 | 1 | | | 0.000*** |
| 70-74 | 2.516(1.495-4.235) | | | 0.001* |
| ≥75 | 3.795(1.715-8.396) | | | 0.001* |
| **Recurrent meningioma (yes/no)** | 7.201(3.054-16.981) | | | 0.000*** |
| **Location** |  |  |  |  |
| Convexity | 1 | | | 0.017* |
| Falx/sagittal sinus | 2.502(1.275-4.911) | | | 0.008* |
| Tentorium | 1.664(0.651-4.253) | | | 0.288 |
| Skull base | 2.780(1.454-5.316) | | | 0.002* |
| Intraventricular | 4.035(0.762-21.365) | | | 0.101 |
| **Maximal diameter, cm(>6/≤6)** | 2.729(1.309-5.689) | | | 0.007* |
| **Nerves involved (yes/no)** | 2.893(1.487-5.628) | | | 0.002* |
| **WHO grade and biological behavior (High risk/Low risk)** | 3.130(1.695-5.778) | | | 0.000*** |
| **Preoperative KPS** | 0.961(0.935-0.986) | | | 0.003* |
| **KPS on discharge** | 0.969(0.952-0.985) | | | 0.000*** |
| C-index：0.8100(95% CI:0.7689-0.8503)  Hosmer-Lemeshow test：χ2=6.081, *P*=0.638  VIF = 1.052-1.175 | | | | |

**P*<0.05;****P*<0.001
